# Molecular and quantitative genetic variation within and between populations of the declining grassland species *Saxifraga granulata*

**DOI:** 10.1002/ece3.9462

**Published:** 2022-11-18

**Authors:** Tania J. Walisch, Guy Colling, Sylvie Hermant, Diethart Matthies

**Affiliations:** ^1^ Musée National d'Histoire Naturelle Luxembourg City Luxembourg; ^2^ Department of Biology Philipps‐Universität Marburg Marburg Germany

**Keywords:** climatic distances, diversifying selection, evolutionary potential, evolvability, formerly common species, genetic drift, heritability, isolation by distance, molecular genetic variation, pairwise *Q*_st_, *Q*_ST_ vs. *F*_ST_, quantitative genetic variation, recent fragmentation, *Saxifraga granulata*

## Abstract

Formerly common plant species are expected to be particularly susceptible to recent habitat fragmentation. We studied the population genetics of 19 recently fragmented *Saxifraga granulata* populations (max. distance 61 km) in Luxembourg and neighboring Germany using RAPD markers and a common garden experiment. We assessed (1) the relationships between plant fitness, quantitative genetic variation, molecular genetic variation, and population size; and (2) the relative importance of genetic drift and selection in shaping genetic variation. Molecular genetic diversity was high but did not correlate with population size, habitat conditions, or plant performance. Genetic differentiation was low (*F*_ST_ = 0.079 ± 0.135), and there was no isolation by distance. Longevity, clonality, and the long‐lived seed bank of *S. granulata* may have prevented strong genetic erosion and genetic differentiation among populations. However, genetic distinctness increased with decreasing genetic diversity indicating that random genetic drift occurred in the studied populations. Quantitative and molecular genetic variations were correlated, and their differentiation (*Q*_ST_ vs. *F*_ST_) among *S. granulata* populations was similar, suggesting that mainly random processes have shaped the quantitative genetic differentiation among populations. However, pairwise quantitative genetic distances increased with geographic and climatic distances, even when adjusted for molecular genetic distances, indicating diversifying selection. Our results indicate that long‐lived clonal species may be buffered at least temporarily against the negative effects of fragmentation. The relationship between quantitative genetic and geographic distance may be a more sensitive indicator of selection than *Q*_ST_–*F*_ST_ differences.

## Graphical abstract

We found low overall genetic differentiation between populations of the formerly common grassland species *Saxifraga granulata*. Molecular genetic diversity of populations was high and did not correlate with population size, habitat conditions, or plant performance. Quantitative and molecular genetic variations were correlated, and their differentiation (*Q*_ST_ vs. *F*_ST_) among populations was similar. The results show that in fragmented species, strong genetic drift and the consequent loss of genetic diversity may be prevented by polyploidy, longevity, and a long‐lived seed bank and that the relationship between quantitative genetic and geographic distance may be a more sensitive indicator of selection than *Q*_ST_−*F*_ST_ differences.

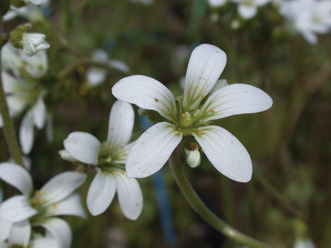

## 1 INTRODUCTION

The intensification of agricultural land use at the expense of traditional land management practices has caused a decline of semi‐natural grasslands in Western Europe (Matthies, 2000; Poschlod et al., 2005), and many formerly common grassland species now occur in smaller and more isolated populations (Oostermeijer et al., 1996; Saunders, 1991). Fragmented populations are more strongly threatened by environmental and demographic stochasticity (Matthies et al., 2004; Young et al., 1996). Fragmentation reduces gene flow and increases genetic drift and inbreeding leading to a loss of genetic variability (genetic erosion, Aguilar et al., 2008; Ellstrand & Elam, 1993; Fischer & Matthies, 1998a; González et al., 2020; Honnay et al., 2007; Van Rossum et al., 2004; Young et al., 1996), a reduction in plant performance (Aguilar et al., 2019; Fischer & Matthies, 1998b; Kéry et al., 2000; Leimu et al., 2006), and a lower evolutionary potential of populations (Weber & Kolb, 2014; Willi et al., 2006). Population fragmentation tends to increase the differentiation among populations through reduced gene flow and genetic drift (Ellstrand & Elam, 1993; Willi et al., 2007). The sensitivity of a species to genetic erosion depends on life‐history traits such as its longevity or clonal growth (Nybom, 2004; van der Meer & Jacquemyn, 2015), ploidy level (Frankham, 2010; van der Meer & Jacquemyn, 2015), breeding system (Aguilar et al., 2008; Leimu et al., 2006), the efficiency of gene flow between populations through seeds and pollen (Ghazoul, 2005), and the longevity of the seed bank (Honnay et al., 2008). Gene flow is often strongly restricted even within plant populations, because of short distance pollination and limited seed dispersal (Scheepens et al., 2012). This can lead to a pattern of isolation by distance within populations, where individuals that grow close to each other are more closely related than random pairs of individuals (Vekemans & Hardy, 2004), and to a reduction in effective population size.

The evolutionary potential of a population depends on the genetic variation of quantitative traits, which are often under selection (Leinonen et al., 2008; Mittell et al., 2015; Reed & Frankham, 2001; Walisch, Colling, et al. 2015). However, the quantitative genetic variability in populations of fragmented species has been studied far less frequently than that of neutral molecular markers (Edwards, 2015; Kramer & Havens, 2009), although the two types of genetic variability are often not related (Reed & Frankham, 2001; Volis et al., 2016). Studying the evolutionary potential of a population to adapt to changing conditions is important in order to assess its chances to persist in the long term and to develop better conservation measures.

The comparison of genetic differentiation in quantitative traits (*Q*_ST_) with that in neutral molecular genetic markers (*F*_ST_) has been used to estimate the relative contributions of drift and selection to the overall genetic variation among populations (Merilä & Crnokrak, 2001). If *Q*_ST_ is similar to *F*_ST_, drift is the major evolutionary force shaping the overall genetic differentiation among populations. If *Q*_ST_ is larger or smaller than *F*_ST_, divergent or stabilizing selection is contributing to the overall genetic variation among populations (Volis et al., 2005; Walisch, Colling, et al. 2015). In most studies, *Q*_ST_ was larger than *F*_ST_ indicating that divergent selection is common in plant populations (e.g., meta‐analyses by Leinonen et al., 2008; De Kort et al., 2013; Walisch, Colling, et al., 2015). However, studies on common or recently fragmented grassland species have obtained conflicting results. In a study of *Scabiosa columbaria* in calcareous grasslands in the Swiss Jura (Scheepens et al., 2010), unifying selection was detected, while in a study in a small geographic area in Sweden, the same species showed signs of divergent selection (Waldmann & Andersson, 1998). In highly fragmented temperate grasslands of Australia, *Rutidosis leptorrhynchoides* showed divergent selection along environmental gradients (Pickup et al., 2012).

**FIGURE 1:**
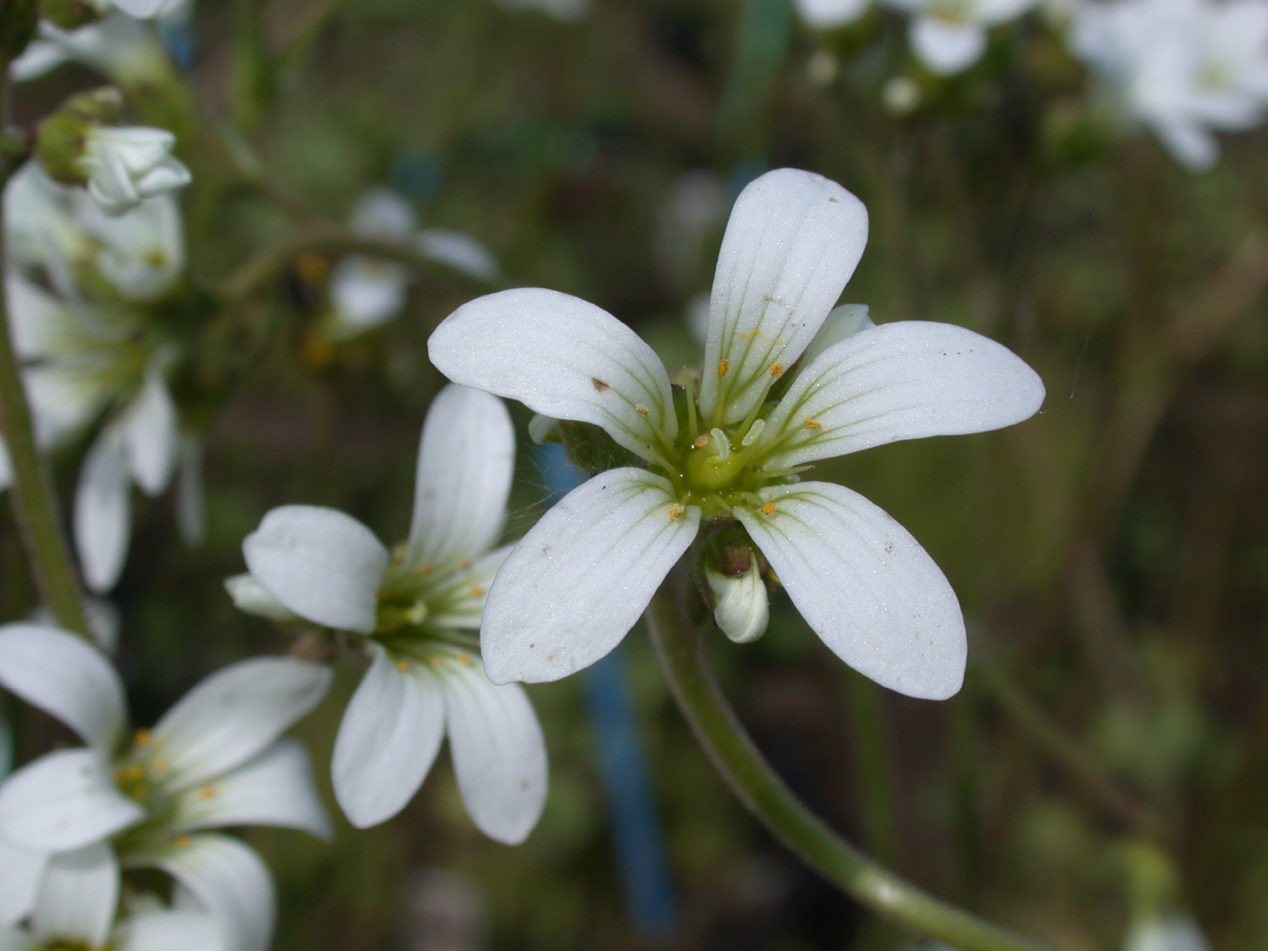
*Saxifraga granulata* L.

We studied the molecular and quantitative genetic variation within and among populations of the grassland species *Saxifraga granulata* (Figure 1) in Luxembourg and a neighboring area in Germany to investigate the effects of the recent fragmentation on its populations. *S. granulata* is a formerly common grassland species that has strongly declined in the last decades and is now threatened in several European regions (Metzing et al., 2018; van der Meer & Jacquemyn, 2015; Walisch et al., 2012). A recent genetic study of *S. granulata* along two rivers in central Belgium found that populations had maintained high molecular genetic diversity despite increasing fragmentation (van der Meer & Jacquemyn, 2015). Our study was conducted in a different habitat, mesic grasslands, and extends the study by including quantitative genetic variation. We addressed the following questions: (1) Are there positive correlations between the performance of plants, quantitative genetic variation, molecular genetic variation, and population size? (2) Are molecular and quantitative genetic differentiation between populations related to geographical distance, and what is the relative importance of selection and drift for genetic differentiation?

## 2 MATERIALS AND METHODS

### 2.1 Study species

*Saxifraga granulata* L. is a perennial herb that is propagated both sexually by seeds and vegetatively by small bulbils produced at the base of the plant (Kaplan, 1995; Stroh, 2015). The seeds are very small (c. 0.5 × 0.3 mm, c. 40 μg) and dispersed by wind. Seedling establishment in the field is very low, and the main means of propagation is thought to be via bulbils (Richards, 1986). The above‐ground parts wither over summer, and a new basal rosette is produced in autumn, which overwinters and may flower the next spring. The flowers of *S. granulata* are protandrous, but self‐compatible (Hansen & Molau, 1994; Walisch et al., 2012). Pollination is assured by a wide range of insect species including flies and solitary bees (Hansen & Molau, 1994). Geitonogamous selfing within the same genet is common. A pollination study in a large population of *S. granulata* in Luxembourg found a mixed mating system with an estimated selfing rate of 55% (Walisch et al., 2012). *Saxifraga granulata* occurs in mesic to dry grasslands across northern, western, and central Europe reaching its southern range limit in North Africa (Stroh, 2015). However, populations have declined over the past decades in many parts of its range (Metzing et al., 2018, van der Meer & Jacquemyn, 2015) due to changes in agricultural practices, such as the increased fertilization of meadows, the conversion of grasslands into arable fields (Walisch et al., 2012), and the use of broad‐spectrum herbicides (Stroh, 2015).

### 2.2 Study sites and collection of samples

In May and June 2002, we selected 15 sites in Luxembourg and four additional ones in the neighboring state of Rheinland‐Pfalz in Germany for a study of the genetic structure of the populations (Table 1). The geographical distance between the sites ranged from 0.05 to 61 km (median: 11). The longitude and latitude of the center of each population were determined with a GPS, and population size was estimated as the number of flowering individuals. For each site, we obtained the bioclimatic variables mean diurnal temperature range, mean annual temperature, temperature seasonality (SD), minimum temperature of the coldest month, maximum temperature of the warmest month, temperature annual range, annual precipitation, precipitation seasonality (CV), precipitation of the wettest month, and precipitation of the driest month at a grid size of about 1 km^2^ (30 arc s) from the Worldclim database version 1.4. (Hijmans et al., 2005). A principal component analysis of climate variables identified two principal components, which explained 93.5% of the total variation. PRECIP explained 76% and correlated strongly with annual precipitation (*r* = .97), precipitation of the driest (*r* = .92) and of the wettest month (*r* = .98), and was negatively correlated with mean diurnal temperature range (*r* = −.81). TEMP explained a further 17.5% of the variance and was highly correlated with the maximum temperature of the warmest month (*r* = .99), annual mean temperature (*r* = .94), and minimum temperature of the coldest month (*r* = .86). Based on the two principle components PRECIP and TEMP, we calculated pairwise climatic distances between populations as Euclidian distances.

**TABLE 1:** Genetic diversity of 19 populations of *Saxifraga granulata*

Region	Population	*N*	*PPL* (%)	*H*_eN_	*N* per family in garden	Spatial reference (lat./long.)
Luxemburg	1	67	94.2	0.338	6.4	N 49.605/E 6.058
	2	8000	92.3	0.378	5.7	N 49.605/E 6.060
	3	59	90.4	0.356	5.0	N 49.609/E 6.026
	4	1000	96.2	0.361	6.0	N 49.597/E 6.067
	5	22,000	98.1	0.348	6.5	N 49.572/E 5.951
	6	2400	96.2	0.364	5.9	N 49.611/E 6.026
	7	3430	84.6	0.327	5.9	N 49.602/E 5.980
	8	11	94.2	0.345	6.0	N 49.614/E 6.018
	9	210	96.2	0.331	6.1	N 49.621/E 6.018
	10	14,800	94.2	0.342	5.6	N 49.5477/E 5.9033
	11	8000	100	0.377	6.0	N 49.5479/E 5.9026
	12	200	94.2	0.350	6.3	N 49.570/E 6.043
	13	5100	92.3	0.339	6.1	N 49.571/E 6.041
	14	15	71.2	0.287	5.8	N 49.490/E 6.001
	15	900	96.2	0.373	6.5	N 49.489/E 6.000
Germany	16	4150	96.2	0.329	5.4	N 49.682/E 6.708
	17	200	96.2	0.347	5.6	N 49.682/E 6.710
	18	2700	90.4	0.302	5.6	N 49.709/E 6.706
	19	5000	90.4	0.357	6.6	N 49.706/E 6.703

*Note*: *N*, number of flowering plants in the population in the year 2002; *PPL*, proportion of polymorphic loci at the 5% level; *H*_eN_, Nei's gene diversity based on allele frequencies calculated with the Bayesian method with nonuniform prior distribution of allele frequencies in a population (Zhivotovsky, 1999) assuming that the inbreeding coefficient *F*_IS_ = 0.643. *N* per family denotes the mean number of plants per family studied in the common garden.

In 2003, we selected 15 plants along a 15 m transect in each population. At two large grassland sites, we sampled two transects in two subpopulations separated by at least 50 m from each other (Table 1, populations 10–11 and 12–13). In most populations, the plants were at least 0.5 m apart in order to minimize the chance of sampling clones. However, in very small populations, sampling distances were smaller. To calculate the distances between the plants, we recorded their relative spatial position along the transect. We counted the number of flowers of each plant. From each plant, we collected 1–2 basal leaves for molecular genetic analyses and one ripe capsule for a common garden experiment. All leaf samples were immediately frozen at −80°C for molecular genetic analysis.

### 2.3 Cultivation of plants

In September 2009, we placed two batches of 15 seeds per mother plant in separate Petri dishes on moist filter paper and stratified them in a growth chamber at 4°C for 4 weeks. The temperature was raised to 20°C at the end of October, and the seeds were put under a 12 h day/12 h night light regime. The position of the Petri dishes was randomized every 3–4 days. Seed germination was recorded every 2 weeks, and 3–10 seedlings per mother plant (hereafter referred to as a seed family) of a minimum size of 1 cm were selected at random and planted into soaked peat pellets (“Jiffy pots”). The plants were placed on trays under fluorescent tubes (Gro‐Lux, 28 W, Osram Sylvania). Early survival of plants was recorded for 2 months. In March 2010, we measured for each plant (*n* = 1521) the largest diameter, the width of the longest leaf and counted the number of flowers. There were on average 14 families per population and 5.7 plants per family.

### 2.4 RAPD‐PCR

The frozen dried leaf material was ground (Retsch MM200, Retsch), and DNA extracted using the DNeasy® Plant Mini Kit (QIAGEN). We carried out amplifications in 25 μl volumes containing 5 μl of template DNA (5 ng DNA/μl), 8.575 μl ddH_2_O, 3 μl MgCl_2_ (25 mM); 0.5 μl dNTP's (10 mM), 2.5 μl PCR Buffer with (NH_4_)_2_SO_4_ (10X, Fermentas); 5 μl Primer (5 μM); 0.3 μl *Taq* DNA Polymerase (5 units/μl, Fermentas); and 0.125 μl BSA (20 mg/mL). The volumes were held in polycarbonate microtitre plates and covered by adhesive sealing sheets. The plates were then incubated in a thermocycler (iCycler®, Bio‐Rad Laboratories) programmed with the following settings: Denaturation of the DNA at 94°C for 2 min, followed by 44 repetitive cycles consisting of denaturation for 45 s at 94°C, annealing for 2 min 30 s at 36°C, and extension for 2 min at 72°C followed by a final extension phase of 5 min at 72°C. The samples were kept at 4°C until analysis. Amplified DNA fragments were separated by electrophoresis on precast ReadyAgarose™ 1.0% Agarose gels with ethidium bromide in 1× TBE buffer (Bio‐Rad Laboratories) in an electrical field (85 V, c. 100 min). The gels were put under UV light and photographed using the Bio Doc system (Bio‐Rad Laboratories).

In a first series of amplifications 60 10‐base primers (Kits A, B, C from Operon Technologies) were screened in a random sequence and tested for reproducibility of the amplified fragment profile using four replicates of a single DNA extract. The first seven primers yielding good‐quality reproducible patterns (primers A4, A7, A11, C1, C2, C6, C8) were selected for the RAPD analysis of 250 sampled plants (Table 2). Presence or absence of reliable bands on amplification products was scored visually using the program Quantity/One (Bio‐Rad Laboratories), which were treated as phenotypes, with each band position representing a character either present or absent. The final presence–absence matrix contained scores at 54 polymorphic band positions for all samples in the study. We replicated 356 combinations of DNA samples and markers after DNA extraction to estimate the error rate of the RAPD genotyping resulting in 2771 repeated banding scores (corresponding to 20.5% of the total dataset). The second scoring was done by the same technician as the first one, and the error rate was estimated to be 6.6%. Because of the error rate of 6.6%, we considered plants differing by up to 3.6 (rounded to 4) loci as putative clones belonging to the same genotype (Ehrich et al., 2008). We only kept one randomly chosen putative clone per genotype in the RAPD matrix resulting in 247 samples used for further analysis.

**TABLE 2:** RAPD primers used

Primer	Sequence
A4	5′‐ AATCGGGCTG‐ 3′
A7	5′‐ GAAACGGGTG‐ 3′
A11	5′‐ CAATCGCCGT‐ 3′
C1	5′‐ TTCGAGCCAG ‐ 3′
C2	5′‐ GTGAGGCGTC ‐ 3′
C6	5′‐ GAACGGACTC ‐ 3′
C8	5′‐ TGGACCGGTG ‐ 3′

We identified markers under divergent or balancing selection with the program BAYESCAN 2.1 with the false discovery rate set to 0.05 (see Foll & Gaggiotti, 2008). Several methods of detecting markers under selection have recently been tested by De Mita et al. (2013). The method used by BAYESCAN was found to be robust against deviations from the island model and yielded very few false positives in all simulations. We removed any markers that were putatively non‐neutral and used the resulting matrix of neutral loci in subsequent analyses.

## 3 DATA ANALYSIS

### 3.1 Molecular genetic diversity within populations and structure among populations

To estimate allele frequencies, we used the Bayesian method with nonuniform prior distribution of allele frequencies (Zhivotovsky, 1999) as implemented in AFLP‐SURV version 1.0 (Vekemans, 2002) with an estimate of Wright's inbreeding coefficient over all populations (*F*_IS_). *F*_IS_ was calculated using the approximate Bayesian computation for *F*‐statistics (ABC4F) for dominant data (Foll et al., 2008). Genetic diversity within populations was calculated as (1) the percentage of polymorphic loci (*PPL*) at the 5% level, and (2) Nei's gene diversity (expected heterozygosity *H*_eN_) according to the method of Lynch and Milligan (1994) which uses the average expected heterozygosity of the marker loci.

The genetic structure among populations was analyzed on the basis of RAPD allele frequencies using AFLP‐SURV assuming the inbreeding coefficient *F*_IS_ calculated by ABC4F. The significance level of the calculated *F*_ST_ and its confidence interval were estimated by 1000 permutations. A pairwise genetic distance matrix with *F*_ST_ values was calculated in AFLP‐SURV using the *F*_IS_‐estimate over all populations. The partitioning of genetic variation among populations and among individuals within populations was investigated by analysis of molecular variance (AMOVA) using GenAlex version 6.501 (Peakall & Smouse, 2006, 2012, see Excoffier et al., 1992, Stewart & Excoffier, 1996).

We also calculated the mean genetic distance between each population and all other populations (mean pairwise *F*_ST_) and related it to the genetic diversity of the populations to test whether genetic drift might have simultaneously resulted in increased distinctness of populations and reduced genetic diversity (see Yakimowski & Eckert, 2008).

### 3.2 Within and between population quantitative genetic variation

We analyzed the effects of population and family nested within population on the measured plant traits. To obtain estimates of between population genetic variation (*Q*_ST_), heritability (*h* ^2^) and evolvability (genetic coefficient of variation, CV_genetic_; Houlé, 1992), we calculated variance components between populations (*V*_pop_), between families within populations (*V*_fam_), and between individuals within families (*V*_error_) for each trait by restricted maximum likelihood with the lmer and VarCorr functions of the R‐package lme4 v.1.1–30 (Bates et al., 2015). Heritability (*h* ^2^) was calculated as $h^{2}=\left(V_{\mathrm{fam}}/2^{*}\theta \right)/\left(V_{\mathrm{fam}}+V_{\text{error}}\right)$, and the evolvability (genetic coefficient of variation) as ${\mathrm{CV}}_{\text{genetic}}=\surd \left(V_{\mathrm{fam}}/2^{*}\theta \right)/\text{mean}$, where *θ* is a measure of the kinship of the plants (see Jimenez‐Ambriz et al., 2007). For selfed plants, the value of *θ* is 0.5 and for full‐sibs 0.25. In a previous pollination study conducted in a large population of *S. granulata* in Luxembourg, the estimated selfing rate was 55% (Walisch et al., 2012). We inferred that 55% of offspring originated from selfing in our study populations and assumed that the remaining 45% of offspring were full‐sibs to obtain a weighted mean value of 0.3875 for *θ*. *Q*_ST_ was hence calculated as $V_{\mathrm{pop}}/\left(\left[V_{\mathrm{fam}}/\theta \right]+V_{\mathrm{pop}}\right)=V_{\mathrm{pop}}/\left(V_{\mathrm{fam}}/0.3875+V_{\mathrm{pop}}\right)$. We calculated 95% confidence intervals of *Q*_ST_ by jackknifing over populations (O'Hara & Merilä, 2005).

We calculated mean CV_genetic_ of all traits over the populations and tested whether they were significantly different from 0 using one sample *t*‐tests. We used simple regressions to explore the relationship between the evolvability (CV_genetic_) of each trait in a population and its heritability (*h* ^*2*^) and its population mean value. We estimated the overall quantitative genetic variability as the mean evolvability over all traits and studied the relation between mean evolvability and expected heterozygosity, *PPL*, mean heritability of the quantitative traits, and population size with simple regressions. We also analyzed the relationship between population mean trait values and heritability.

We tested the relationship between pairwise molecular genetic (*F*_ST_) and pairwise geographic distances between populations with the permutation test available in the R‐package lmPerm (Wheeler & Torchiano, 2016). We calculated quantitative genetic distances between pairs of populations as Mahanalobis distances based on plant diameter, leaf width, and number of flowers averaged over families. Mahanalobis distances measure distances in multivariate space taking into account correlations among traits and are independent of the scale of the traits (Legendre & Legendre, 1998). To test for isolation by distance for the quantitative traits, we related pairwise Mahanalobis distances to geographic distances and tested its significance with a permutation test (R‐package lmPerm). We also tested the relationship between pairwise quantitative genetic and molecular genetic distances, climatic distances, and geographic distances between populations with a sequential model in lmPerm. In this model, effects of each variable were thus adjusted for those of the variables preceding it. All statistical analyses, if not stated otherwise, were carried out with SPSS 25.0 (IBM Corp., 2017).

## 4 RESULTS

### 4.1 Molecular genetic diversity and population structure

**FIGURE 2:**
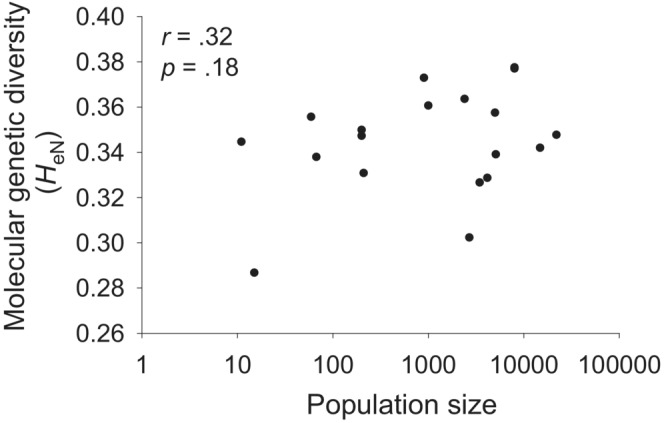
The relationship between Nei's gene diversity, *H*_eN_, and the size of a population.

The seven RAPD primers used for analysis generated a total of 54 polymorphic bands. No private (population‐specific) bands were observed, and each individual had a unique band pattern. Considering the error rate of 6.6%, three pairs of individuals, which differed in less than five bands, were considered as putative clones, and one of each pair was removed at random. The distance between members of the same putative clone ranged from 0.01 to 0.93 m. We removed two bands that had been identified as putative non‐neutral loci (C02F and C02G; 4% of all loci) by the program BAYESCAN 2.1 and obtained a final matrix of 247 unique genotypes and 52 neutral loci for our study populations. The mean proportion of polymorphic loci (*PPL*) in the 19 populations was 92.8% and varied among the populations from 71.2% to 100% (Table 1). Mean Nei's gene diversity (*H*_eN_) per population using the *F*_IS_ estimated by ABC4F (*F*_IS_ [*f*] = 0.643 ± 0.04) was 0.345 and varied from 0.287 in the very small population Lallange to 0.378 in the large population Niedercorn (Table 1). Molecular genetic diversity increased with population size, but the relationship was weak and not significant (*r* = .32, *p* = .18, Figure 2).

**FIGURE 3:**
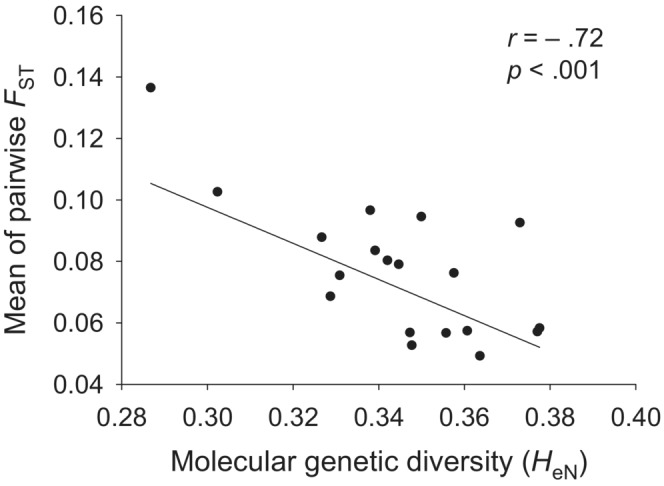
The relationship of the mean *F*_ST_ between each population and all other populations and Nei's gene diversity (*H*_eN_) of the populations.

The AMOVA analysis showed that 11% of the variation was among populations (*p* < .001), while variation among individuals within populations accounted for 89%. *F*_ST_ estimated by AFLP‐SURV (assuming *F*_IS_ = 0.643) was 0.079 ± 0.1348. The mean *F*_ST_ between a population and all other populations was negatively related to its genetic diversity, i.e., the lower the molecular genetic diversity of a population was, the more distinct was it (*r* = −.72, *p* < .001; Figure 3).

### 4.2 Population performance and quantitative genetic variation

There was no significant relationship between the various measures of performance in the common garden and the size of the population of origin or its molecular genetic diversity (Table 3). The mean number of flowers produced per plant in the common garden and in the population of origin was only very weakly correlated (*r* = .14, *p* = .56).

**TABLE 3:** Correlations between various performance measures of *Saxifraga granulata* plants raised in the common garden and the size and molecular genetic diversity of their population of origin.

Trait	Population size		*H*_eN_	
	*r*	*p*	*r*	*p*
Germination	.05	.852	.12	.621
Early mortality	−.03	.913	.15	.545
Plant diameter	.24	.319	−.19	.446
Leaf width	.23	.345	−.06	.819
No. of flowers (sqrt‐transformed)	.23	.339	−.28	.240

**FIGURE 4:**
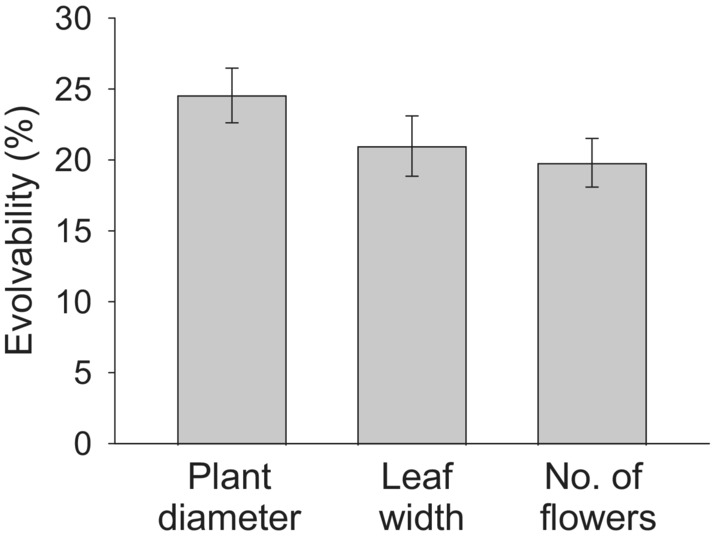
Evolvability of performance traits of *Saxifraga granulata*. Means ± 1 SE.

Mean quantitative genetic diversity within populations estimated as evolvability (CV_genetic_) was significantly larger than zero for plant diameter (*t*_18_ = 12.7, *p* < .001), leaf width (*t*_18_ = 9.9, *p* < .001), and number of flowers (*t*_18_ = 11.5, *p* < .001; Figure 4). Mean evolvability averaged over the studied traits in a population varied from 9% to 31%. Evolvability and heritability (*h* ^*2*^) of the individual traits per population were strongly correlated (all *r* > .87, all *p* < .001), and mean evolvability and mean heritability averaged over all studied traits were also strongly correlated (*r* = .89, *p* < .001).

**FIGURE 5:**
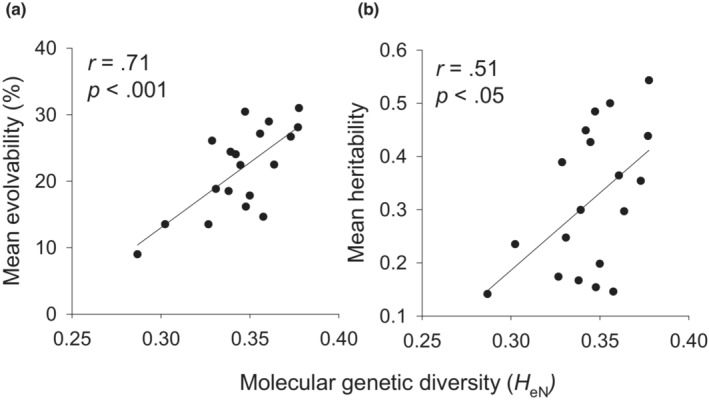
Relationship between (a) mean evolvability and (b) mean heritability of the measured quantitative traits in a population and its molecular genetic diversity (Nei's gene diversity).

**FIGURE 6:**
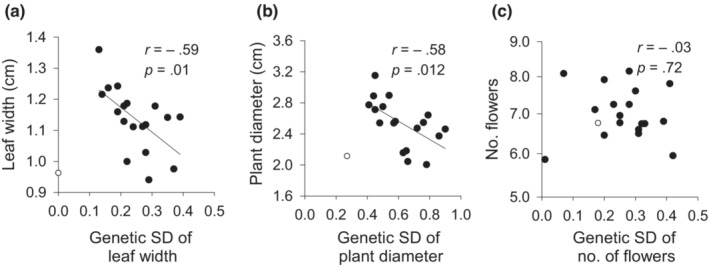
Relationships between the mean and the additive genetic standard deviation of (a) leaf width, (b) plant diameter, and (c) number of flowers per plant in populations of *Saxifraga granulata*. The population Lallange 1 (open circle) was omitted as an outlier from the statistical analyses because it was only recently founded. Note square‐root scale for number of flowers.

There was a strong positive relationship between the mean evolvability over all traits and *H*_eN_ (*r* = .71, *p* < .001; Figure 5a) or *PPL* (*r* = .61, *p* < .01) in a population, as well as between heritability and *H*_eN_ (*r* = .51, *p* < .05; Figure 5b). However, mean evolvability was only weakly related to the size of the population of origin, and this relationship was not significant (*r* = .16, *p* = .514). To relate population means of the quantitative traits with their genetic variation, we related trait means to the standard deviation of genetic variability instead of evolvability (mean‐scaled genetic variance) to avoid spurious correlations. Population means and the standard deviation of genetic variability of plant diameter and leaf width (Figure 6a,b), but not of flower number, were negatively correlated (Figure 6c). None of the trait means correlated significantly with molecular genetic diversity (|*r*| < .28; all *p* > .24).

**FIGURE 7:**
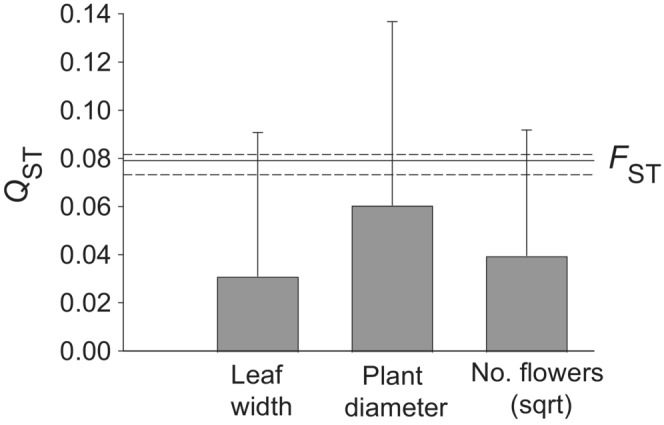
Molecular genetic (*F*_ST_) and quantitative genetic differentiation (*Q*_ST_) between populations of *Saxifraga granulata*. Bars show 95% confidence intervals of *Q*_ST_ and dashed lines those of *F*_ST_.

**FIGURE 8:**
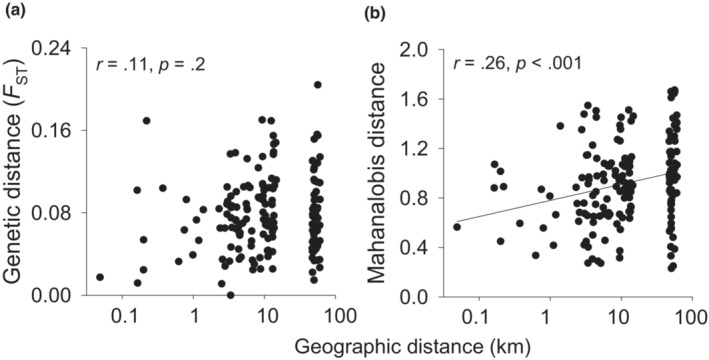
Relationship between the geographic distances between populations and (a) pairwise molecular genetic distances (pairwise *F*_ST_) and (b) pairwise quantitative genetic distances (Mahanalobis distances) between populations.

The quantitative genetic differentiation among populations estimated as *Q*_ST_ was similar to the molecular differentiation (*F*_ST_) for all three quantitative traits studied (Figure 7). The pairwise molecular genetic and geographic distances between populations were not correlated (Figure 8a, *r* =.113, *p* = .14) indicating the absence of an isolation by distance pattern. In contrast, the pairwise quantitative genetic distances increased with geographic distances (Figure 8b, *r* = .26, *p* < .001). This relationship remained strong if corrected for the effect of molecular distance (*p* < .001). Adding the climatic distance between populations in addition to molecular distance had a strong effect (*p* = .008) and explained a large part of the effect of geographical distance on quantitative genetic distance, but geographical distance still had an effect (*p* = .04). These results suggest that the variation in quantitative traits is not determined by random processes alone, but is also a result of local adaptation to different climates and local environmental factors.

## 5 DISCUSSION

### 5.1 Genetic diversity of populations and plant performance

The overall molecular genetic diversity *H*_eN_ of the populations of *S. granulata* (0.35) as estimated by our study was high in comparison to the mean *H*_eN_ found in other RAPD studies (0.22, Nybom, 2004), in other long‐lived perennials (*H*_eN_ = 0.25, Nybom, 2004) or for other species with a mixed mating system (*H*_eN_ = 0.18). Considering that genetic diversities estimated by dominant markers are on average about only a third of that estimated by microsatellite marker studies (Nybom, 2004), the genetic diversity of our study populations was higher than the genetic diversity of riparian *S. granulata* populations in Belgium (*H*_s_ = 0.68) estimated by microsatellite markers (van der Meer & Jacquemyn, 2015). In contrast to many other studies, we did not find reduced genetic diversity in small populations as a sign of drift (Aguilar et al., 2019; Fischer & Matthies, 1998a; Leimu et al., 2006). The recent fragmentation of their habitats due to the intensification of agriculture in the last decades has thus apparently not yet affected the genetic diversity of small *S. granulata* populations. A recent meta‐analysis found in general negative effects of fragmentation on the genetic diversity of populations isolated for more than 50 years, but not for those isolated more recently (Schlaepfer et al., 2018). Similar to *S. granulata*, several other perennial long‐lived species of European grasslands also showed a lack of a correlation between population size and genetic diversity, including *Scabiosa columbaria* (Waldmann & Andersson, 1998), *Primula veris*, *Dianthus carthusianorum, Medicago falcata, Polygala comosa*, and *Salvia pratensis* (Reisch et al., 2017). The long‐lived seed bank (Milberg, 1992) and the longevity and clonality of the *S. granulata* plants may have buffered populations against genetic erosion (Nybom, 2004; van der Meer & Jacquemyn, 2015). A further reason for the high overall genetic diversity of *S. granulata* could be its polyploidy. As polyploidy plants contain more copies of the genome, they have a higher potential for mutations, and they are less prone to drift than diploids (Meirmans & Van Tienderen, 2013; van der Meer & Jacquemyn, 2015).

Inbreeding has very strong negative effects on the performance of *S. granulata* (Walisch et al., 2012). However, we found no relationship between plant performance in a common garden and the molecular genetic diversity or size of the population of origin, indicating no inbreeding depression in small populations. This is in contrast to the negative effects of fragmentation on the performance of other grassland plants (Bowman et al., 2008; Busch & Reisch, 2005; Fischer & Matthies, 1998b; Kéry et al., 2000; Schleuning et al., 2009; Vergeer et al., 2003). The lack of a relation between plant performance and molecular genetic diversity in the study populations could be due to the lack of genetic erosion in small populations, which restricted the range of genetic diversity observed (0.29–0.38).

### 5.2 Molecular genetic variation between populations

The level of differentiation between the fragmented *S. granulata* populations was low (*F*_ST_ = 0.11) and was much lower than the mean Φ_ST_ found in studies of species with a similar life history using dominant markers (mixed mating species *Φ*_ST_ = 0.40, long‐lived species *Φ*_ST_ = 0.25; Nybom, 2004). Our *F*_ST_ value was also lower than the *Φ*_ST_ values obtained in other studies at a similar geographical scale (e.g., Allnutt et al., 1999; Colling et al., 2010; Kuss et al., 2008; Müller et al., 2012; Tollefsrud et al., 1998). Similarly low levels of genetic variation among populations have been found in *S. granulata* along two river systems in Belgium (*G*_ST_ = 0.093–0.042; van der Meer & Jacquemyn, 2015, maximum distance c. 60 km) and in *Pimelea spinescens* in fragmented temperate grasslands in Southeast Australia (*F*_ST_ = 0.07, James & Jordan, 2014).

Possible reasons for the low differentiation between populations are the same as for the high genetic diversity: extensive gene flow in the past due to a high connectivity of populations, and the preservation of this historic genetic pattern over time in spite of recent fragmentation due to the longevity of plants and polyploidy. The small maximum distance between the sampled populations (61 km) may also have contributed to the low variation among populations, as *F*_ST_ tends to increase with the distance between populations (Crispo & Hendry, 2005; Garnier et al., 2004; Kuss et al., 2008; Nybom, 2004). In contrast, current gene flow between populations is unlikely to have contributed to the low differentiation, because the distance between the great majority of the studied populations was more than 5 km. Low genetic distances between populations of *S. granulata* have also been found in a study of riparian populations in Belgium and attributed to the same factors (van der Meer & Jacquemyn, 2015). However, in that study the maximum genetic distance between populations was much higher, in spite of similar maximum geographic distances. Moreover, in contrast to our study, the Belgian populations showed a pattern of IBD in both studied river systems, indicating moderate gene flow between populations, particularly between those that are close to each other (van der Meer & Jacquemyn, 2015). The lack of IBD in our study could be due to more extensive gene flow in the past unrelated to distance in the study region.

Although mean *F*_ST_ in *S. granulata* was relatively low, we found also signs of genetic drift, as the mean genetic distance between a population and all other populations (distinctness) was negatively related to its genetic diversity, indicating that the higher the loss of genetic diversity through drift was, the more distinct became a population. A similar relationship has been found in *Vaccinium stamineum* (Yakimowski & Eckert, 2008), *Saxifraga sponhemica* (Walisch, Matthies, et al., 2015), and *Gladiolus palustris* (Daco et al., 2019).

### 5.3 Quantitative genetic variation

Quantitative genetic diversity as measured by mean evolvability or heritability of the studied traits was not affected by population size, suggesting that genetic drift has not decreased the adaptive potential of small populations of *S. granulata*. This result is in line with the conclusions of a meta‐analysis by Wood et al. (2016), who found no relation between heritability (*h* ^2^) and population size.

We found a positive relation between the evolvability and the heritability (*h* ^*2*^) of each trait and averaged over all studied traits in a population, which is in contrast to the general conclusions of reviews by Houlé (1992) and Hansen et al. (2011) that evolvability and heritability are generally not correlated. Genetic variation of leaf width and plant diameter was negatively correlated with their population means observed in the common garden, indicating that the genetic variation in growth traits has been much reduced in populations where there has been strong selection for fast growth. This is in line with quantitative genetic theory that the selection of a fitter larger phenotype due to selection may go hand in hand with a loss of quantitative genetic variability within populations (Bulmer, 1971; Visscher et al., 2008).

Quantitative genetic variation of the populations of *S. granulata* measured as both evolvability and heritability was significantly correlated with molecular genetic diversity, in contrast to the results of many other studies (see review of Leinonen et al., 2008; Mittell et al., 2015; Reed & Frankham, 2001; Walisch, Colling, et al., 2015, but see Toczydlowski & Waller, 2021). Both strong divergent and stabilizing selection will reduce the correlation between quantitative and molecular genetic variation, which is thus usually very low (Reed & Frankham, 2003). In contrast, the observed correlation in *S. granulata* indicated that there was no general strong effect of stabilizing or divergent selection on quantitative genetic variation.

Differentiation in quantitative genetic traits among populations of *S. granulata* as measured by *Q*_ST_ was similar to that in molecular genetic variation as indicated by *F*_ST_ and thus provided no evidence for an effect of selection on differentiation in quantitative traits (Merilä & Crnokrak, 2001). This is in contrast to the results of most quantitative genetic studies, which reported divergent selection (De Kort et al., 2013; Leinonen et al., 2008, 2013; Merilä & Crnokrak, 2001). The studied populations originated from a relatively small region and have similar habitats with limited variation of local environmental conditions, which have not resulted in an increase in mean quantitative genetic variation over the level expected by drift. However, in contrast to differentiation in neutral molecular genetic markers, differentiation in quantitative genetic variation increased with geographic distance in *S. granulata*, even when the potential effects of drift were controlled for, indicating divergent selection. Part of the relationship between quantitative genetic differentiation and geographic distance could be explained by increasing differences in climate, suggesting adaptation of the populations of *S. granulata* to local climatic conditions. However, after adjusting for the effects of climatic distance, quantitative genetic differentiation still increased with geographic distance indicating that differences in other environmental factors that increase with geographic distance, e.g., soil conditions, have also contributed to genetic differences between populations.

## 6 CONCLUSIONS

The populations of *S. granulata* in the study area have been fragmented in the last decades, but the low overall genetic differentiation between populations, the similar levels of genetic variation in small and large populations, and the lack of evidence for a reduced fitness of plants from small populations due to drift load indicate that this formerly common species has not yet suffered from the fragmentation of its habitat. Strong genetic drift and the consequent loss of genetic diversity may have been prevented by polyploidy a long‐lived seed bank and longevity of genets due to the production of vegetatively produced bulbils. Clonal growth makes genets potentially immortal and represents a potent buffer against the loss of diversity in populations (Eriksson, 1993; Watkinson & White, 1986). However, we also found signs of genetic drift, and extant populations are thus likely to be affected by ongoing fragmentation in the future. Conservation management should aim to preserve the current populations, increase the size of small populations, and reduce their isolation.

*Q*_ST_−*F*_ST_ comparisons provided no evidence for effects of selection on genetic differentiation in quantitative traits among populations. However, a pattern of isolation by distance for the quantitative genetic variation indicated diversifying selection and possible local adaptation of the populations. The contrasting results obtained by the *Q*_ST_−*F*_ST_ comparison and the correlation between pairwise *Q*_ST_ and geographical distance between populations suggest that an increasing differentiation between populations with geographical distance in quantitative traits may be a more sensitive indicator of divergent selection than comparisons between *Q*_ST_ and *F*_ST_. The significant correlation between evolvability of quantitative traits and molecular genetic variation suggests that in studies on a small geographical scale and in similar environments, molecular genetic diversity could be a useful measure of the evolutionary potential of populations.

## AUTHOR CONTRIBUTIONS

**Tania Josée Walisch:** Conceptualization (equal); data curation (lead); formal analysis (equal); investigation (equal); methodology (equal); writing – original draft (lead). **Guy Colling:** Conceptualization (equal); formal analysis (equal); methodology (equal); supervision (equal); writing – review and editing (equal). **Sylvie Hermant:** Data curation (supporting); investigation (equal); methodology (supporting). **Diethart Matthies:** Conceptualization (equal); formal analysis (lead); methodology (equal); supervision (lead); writing – review and editing (lead).

## CONFLICT OF INTEREST

None declared.

### OPEN RESEARCH BADGES

This article has earned an Open Data badge for making publicly available the digitally‐shareable data necessary to reproduce the reported results. The data is available at [https://doi.org/10.5061/dryad.b8gtht7g5].

## Data Availability

Individual genotype and common garden data as well as population level data are available at https://doi.org/10.5061/dryad.b8gtht7g5.
